# Patient-reported measures in substance use disorder treatment services: a scoping review and preliminary results from a multicenter study

**DOI:** 10.1192/j.eurpsy.2025.311

**Published:** 2025-08-26

**Authors:** C. Migchels, W. van den Brink, A. Zerrouk, W. Vanderplasschen, C. L. Crunelle

**Affiliations:** 1 Psychiatry, Vrije Universiteit Brussel, Universitair Ziekenhuis Brussel, Brussels, Belgium; 2 Psychiatry, Amsterdam University Medical Centers, University of Amsterdam, Amsterdam, Netherlands; 3 Special Needs Education, Universiteit Gent, Gent, Belgium

## Abstract

**Introduction:**

Patient-centered treatment and care is a key quality standard within substance use disorder (SUD) treatment services. Patient-Reported Outcome and Experience Measures (PROMs and PREMs) allow us to collect direct feedback from patients on how they perceive health outcomes and quality of care in a systematic way.

**Objectives:**

To identify current practices regarding the use of PROMs and PREMs in clinical practice in SUD treatment services and to develop an electronic self-report tool for routine assessment of PROMs and PREMs in SUD treatment services in Belgium.

**Methods:**

We present results from a scoping review, identifying studies reporting on the use and routine implementation of PROMs and PREMs in SUD services. Additionally, preliminary results from a naturalistic longitudinal multicenter study assessing self-reported sociodemographic characteristics, clinical factors, PROMs, and PREMs in N=189 adults who recently started treatment for SUD in various treatment modalities are presented: the OMER-BE study (Outcome Measurement and Evaluation as a Routine practice in alcohol and other drug services in Belgium).

**Results:**

There is an increasing use of patient-reported measures in SUD services. However, there is large variation in the patient-reported measures that are used, how they are developed, and how and when patient-reported data are collected. The most important barriers and facilitators to the implementation of PROMs and PREMs in clinical practice include burden to and involvement of staff, and leadership and technical support. Alcohol and cocaine were the most commonly used substances among participants of the OMER-BE study, with 59.7% of participants reporting polysubstance use. The 45-, 90-, and 180-day follow-up assessments were completed by 64%, 59% and 54% of participants respectively. At 180-day follow-up, 56% of respondents were still in treatment for SUD.

**Conclusions:**

Guidance is needed to support clinicians in selecting and implementing valid, meaningful, and comparable patient-reported measures to understand and benefit from the impact that PROMs and PREMs can have on treatment quality and outcome. The OMER-BE study provides an example of the insights that can be gained into patient needs through the use of an electronic self-report tool assessing PROMs and PREMs.

**Disclosure of Interest:**

C. Migchels: None Declared, W. van den Brink Consultant of: Wim van den Brink reports a consulting/advisory relationship with Takeda Pharmaceutical Company Limited, Camurus AB, and Clearmind Medicine., A. Zerrouk: None Declared, W. Vanderplasschen: None Declared, C. Crunelle: None Declared

